# Dysregulation of matricellular proteins is an early signature of pathology in laminin-deficient muscular dystrophy

**DOI:** 10.1186/2044-5040-4-14

**Published:** 2014-07-02

**Authors:** Thomas Mehuron, Ajay Kumar, Lina Duarte, Jenny Yamauchi, Anthony Accorsi, Mahasweta Girgenrath

**Affiliations:** 1 Department of Health Sciences, Boston University, 635 Commonwealth Avenue, Boston, MA 02215, USA

**Keywords:** Apoptosis, CMD, ECM, Fibrosis, Inflammation, Matricellular, MDC1A

## Abstract

**Background:**

MDC1A is a congenital neuromuscular disorder with developmentally complex and progressive pathologies that results from a deficiency in the protein laminin α_2_. MDC1A is associated with a multitude of pathologies, including increased apoptosis, inflammation and fibrosis. In order to assess and treat a complicated disease such as MDC1A, we must understand the natural history of the disease so that we can identify early disease drivers and pinpoint critical time periods for implementing potential therapies.

**Results:**

We found that *DyW* mice show significantly impaired myogenesis and high levels of apoptosis as early as postnatal week 1. We also saw a surge of inflammatory response at the first week, marked by high levels of infiltrating macrophages, nuclear factor κB activation, osteopontin expression and overexpression of inflammatory cytokines. Fibrosis markers and related pathways were also observed to be elevated throughout early postnatal development in these mice, including periostin, collagen and fibronectin gene expression, as well as transforming growth factor β signaling. Interestingly, fibronectin was found to be the predominant fibrous protein of the extracellular matrix in early postnatal development. Lastly, we observed upregulation in various genes related to angiotensin signaling.

**Methods:**

We sought out to examine the dysregulation of various pathways throughout early development (postnatal weeks 1-4) in the *DyW* mouse, the most commonly used mouse model of laminin-deficient muscular dystrophy. Muscle function tests (stand-ups and retractions) as well as gene (qRT-PCR) and protein levels (western blot, ELISA), histology (H&E, picrosirius red staining) and immunohistochemistry (fibronectin, TUNEL assay) were used to assess dysregulation of matricelluar protieins.

**Conclusions:**

Our results implicate the involvement of multiple signaling pathways in driving the earliest stages of pathology in *DyW* mice. As opposed to classical dystrophies, such as Duchenne muscular dystrophy, the dysregulation of various matricellular proteins appears to be a distinct feature of the early progression of DyW pathology. On the basis of our results, we believe that therapies that may reduce apoptosis and stabilize the homeostasis of extracellular matrix proteins may have increased efficacy if started at a very early age.

## Background

The congenital muscular dystrophies (CMDs) are a highly heterogeneous group of degenerative neuromuscular disorders that arise primarily from defects in proteins within or relating to the dystrophin–glycoprotein complex (DGC) [1,2]. Merosin-deficient CMD type 1A (MDC1A) results from a mutation in the α_2_ chain of laminin 211, an isoform essential to skeletal muscle which is responsible for aiding membrane stability and signal transduction across the sarcolemma [3]. Absence of a functional copy of this protein results in severe dysregulation of a wide range of signaling pathways, ultimately leading to apoptosis, failed regeneration, chronic inflammation, fibrosis and muscle wasting [4-8]. The effects manifest so early in life that patients with MDC1A present with profound muscle weakness and hypotonia, either at or soon after birth, and rarely achieve independent ambulation [9-11]. Children with this disease typically die as a result of respiratory complications or failure to thrive by their early teens [12]. Although great strides have been made in research, no effective therapies or treatments are available to patients with MDC1A.

One of the greatest problems researchers face is an essential lack of understanding of how the various pathologies of this disease progress early in life. Because this is a multifaceted, aggressive illness, causative and correlative pathomechanisms must be discriminated at early time points in order for effective treatments to be developed. At this point, only a few studies have been conducted in humans and in animal models of MDC1A at very early time points in attempts to elucidate the early pathomechanisms of this disease [13-18], and no extensive natural history of MDC1A has been documented at a molecular level. Although quite a few researchers have tested the efficacy of various drugs and transgenic therapies in models of MDC1A, their reasoning for choosing those therapies often must be extrapolated from research on Duchenne muscular dystrophy (DMD). However, these two diseases have distinct defects (one results from a defect in an intracellular protein, the other from an extracellular one) and must be treated as such in the context of understanding the pathomechanisms driving each disorder [19,20].

It has previously been demonstrated that patients with MDC1A have stunted growth, limited regenerative capacity and an early surge of inflammation followed by extensive fibrosis in the months soon after birth [18,21]. In our present study, we used the *Lama2*^*DyW*^ (*DyW*) mouse model to recapitulate the human disease progression and show that each of these pathologies, though consistently present through postnatal development, are dynamic and evolve in complexity over time. Using histological and biochemical analyses at postnatal weeks 1, 2, 3 and 4, we observed that the muscles of these mice exhibited very early signs of elevated apoptosis and impaired myogenesis as early as week 1. Most notably, we saw that the expression of various matricellular proteins was dysregulated at the first week after birth, which may help to shed light on the nature of inflammation and fibrosis in this disease. We believe that those pathways causing the dysregulatation of extracellular proteins earliest in development are part of the etiology of the rapid progression of this disease and may serve as the most relevant targets for early intervention in MDC1A.

## Methods

### Animal breeding and care

All animals were housed at the Laboratory Animal Care Charles River Campus of Boston University on a 12:12-hour light–dark cycle. Food and water were provided *ad libitum*. All procedures were performed in accordance to the protocol approved by the Institutional Animal Care and Use Committee of Boston University. Heterozygous B6.129 *Lama2*^*dy-W/+*^ (*DyW*) mice carrying a targeted mutation in the *Lama2* gene were kindly provided by Dr Eva Engvall (Burnham Institute, La Jolla, CA, USA). Of the available mouse models, the *DyW* mouse is the most widely used for studying MDC1A pathology.

### Muscle tissue collection

Animals were euthanized with isofluorane (Webster Veterinary, Devens, MA, USA) before isolating the tibialis anterior (TA), gastrocnemius–soleus complex (GS) and quadriceps muscles (QD). Tissues were weighed and snap-frozen in liquid nitrogen for RNA and protein extraction. TA muscles used for histology were embedded in Tissue-Tek OCT compound (Sakura Finetek USA, Torrance, CA, USA) and frozen in isopentane (Sigma-Aldrich, St Louis, MO, USA) chilled in liquid nitrogen. Serial transverse sections (7 μm) were prepared using the Leica CM1850 cryostat (Leica Microsystems, Buffalo Grove, IL, USA) and stored at -80°C.

### Muscle histology

Frozen sections were air-dried at room temperature for 15 minutes and fixed in chilled acetone for 5 minutes. Sections were hydrated through decreasing grades of alcohol. Midbelly cross-sections were stained with hematoxylin (Fisher Scientific, Fair Lawn, NJ, USA) for 1 minute, followed by development in 1% ammonium hydroxide for 1 minute. Sections were subsequently stained with Ruben’s Eosin-Phloxine working solution (Fisher Scientific) for 2 minutes. After dehydration through increasing grades of alcohol and xylene, sections were mounted using Permount mounting medium (Fisher Scientific). Picro-Sirius Red (American MasterTech Scientific, Lodi, CA, USA) staining of the sections, which had been fixed with acetone and rehydrated was done according to the manufacturer’s instructions. Briefly, the sections were stained with Picro-Sirius Red solution for 15 minutes, subsequently rinsed twice in 0.5% acetic acid and then dehydrated with increasing grades of alcohol and mounted in Permount medium.

A Nikon DS-Fi1 camera head attached to a Nikon ECLIPSE 50*i* light microscope system (Nikon Instruments, Melville, NY, USA) was used to capture images of stained sections. Morphometric analyses were performed using NIS-Elements Basic Research 3.0 software. Myofiber number and cross-sectional area were measured.

### Immunohistochemistry

Frozen tissue sections were fixed in 2% paraformaldehyde for 10 minutes, blocked for 60 minutes with 2% bovine serum albumin, 2% goat serum and 0.1% Triton X-100 in 1× phosphate-buffered saline (PBS). For the Mac-1 stain, slides were incubated with anti-CD11b antibody (1:200 dilution; BD Biosciences, San Jose, CA, USA) for 60 minutes in the dark. For the fibronectin stain, slides were incubated for 2 hours in anti-fibronectin antibody (catalog no. F7387; Sigma-Aldrich) and then incubated in Alexa Fluor 488 goat anti-mouse secondary antibodies in the dark for an additional hour. Nuclei for both assays were stained with 0.1 μg/ml 4′,6-diamidino-2-phenylindole (DAPI) for 5 minutes. After washing with PBS, sections were mounted with VECTASHIELD mounting medium (catalog no. H1000; Vector Laboratories, Burlingame, CA, USA).

### TUNEL assay

Terminal deoxynucleotidyl transferase 2′-deoxyuridine-5′-triphosphate nick-end labeling (TUNEL) staining of the frozen muscle sections was done using ApopTag Plus Fluorescein In Situ Apoptosis Detection Kit (catalog no. S7111; EMD Millipore, Billerica, MA, USA;) as per the manufacturer’s instructions. Briefly, the tissue sections were fixed in 1% paraformaldehyde and subsequently in acetic acid:ethyl alcohol (1:2 dilution) for 5 minutes each. The terminal deoxynucleotidyl transferase labeling was done for 60 minutes at 37°C, which was followed by staining with anti-digoxigenin fluorescein antibody for 30 minutes at room temperature. The sections were washed five times, for 2 minutes each time, in PBS. Nuclear staining was done with 0.1 μg/ml DAPI for 5 minutes. The sections were washed twice as described above and mounted in VECTASHIELD mounting medium. The sections were imaged using the Nikon ECLIPSE 50*i* microscope system equipped with the Nikon DS-Fi1 camera head. Images were analyzed using Nikon NIS-Elements Basic Research 3.0 software.

### Gene expression assay

RNA from 25 mg of snap-frozen hindlimb muscle tissues was extracted with TRIzol reagent (catalog no. 15596-026; Invitrogen, Carlsbad, CA, USA) according to the manufacturer’s instructions. RNA (1 μg) was reverse-transcribed with the High-Capacity cDNA Reverse Transcription Kit (catalog no. 4368814; Applied Biosystems, Foster City, CA, USA). Gene expression analysis was carried out using TaqMan gene expression assays (Applied Biosystems, Foster City, CA, USA) on the Applied Biosystems 7300 Real-Time PCR System. 18S ribosomal subunit RNA served as the endogenous control, and gene expression was calculated by using the comparative ΔΔCt threshold cycle method.

### Western blot analysis

Five biological samples per animal group were pooled for protein extraction. Muscle lysates were prepared by homogenizing 25 mg of muscle in radioimmunoprecipitation assay buffer containing a cOmplete Protease Inhibitor Cocktail tablet (catalog no. 04 693 195 001; Roche Diagnostics GmbH, Mannheim, Germany) and a PhosSTOP Phosphatase Inhibitor Cocktail tablet (catalog no. 04 906 837 001; Roche Diagnostics GmbH). Protein concentration was estimated by Bio-Rad DC Protein Assay (catalog no. 500-0114-6; Bio-Rad Laboratories, Hercules, CA, USA), and 35 μg of protein were resolved on a 10% SDS-PAGE gel and transferred onto a nitrocellulose membrane by semidry electrophoretic transfer (Trans-Blot SD; Bio-Rad Laboratories). Membranes were blocked with Odyssey blocking buffer (catalog no. 927-40000; LI-COR Biosciences, Lincoln, NE, USA) and probed with primary antibodies (phosphorylated Smad2/3 (phospho-Smad2/3), catalog no. 9963; total p65, catalog no. 4764; PhosP65, catalog no. 3033; Cell Signaling Technology, Danvers, MA, USA; Smad7, catalog no. MAB2029; R&D Systems, Minneapolis, MN, USA) and 1:5,000 anti-α-tubulin mouse primary antibodies (catalog no. T9822; Sigma-Aldrich) at 4°C overnight. The blot was washed three times for 5 minutes each time with Tris-buffered saline containing 0.1% Tween 20 and subsequently stained with 1:2,000 goat anti-rabbit IRDye 800CW IgG2b and IRDye 680LT IgG2b antibodies (catalog nos. 926-32352 and 926-68052, respectively; LI-COR Biosciences). The blots were then washed and scanned for analysis using an Odyssey infrared imaging system (LI-COR Biosciences). Each Western blot experiment was repeated three times.

### Hydroxyproline assay

Collagen content in the muscles was determined using a hydroxyproline colorimetric assay kit (catalog no. K555-100; BioVison, Milpitas, CA, USA) according to the manufacturer’s instructions. Briefly, 25 mg of hindlimb muscle tissues from *DyW* and wild-type (*WT*) animals (*n* = 5) were pooled and extracted in 500 μl of distilled water. The lysates were acid-hydrolyzed with HCl (13 M) at 120°C for 3 hours. Equal amounts of lysates and a series of hydroxyproline standards were dispensed in 96-well enzyme-linked immunosorbent assay (ELISA) plates, evaporated at 60°C and oxidized using perchloric acid. The amount of hydroxyproline in samples was detected by chromogenic reaction employing chloramine-T and 4-dimethylaminobenzaldehyde substrate. The absorbance was read at 560 nm.

### Cytokine enzyme-linked immunosorbent assay

The Amount of cytokines in the muscles was determined using ELISA kits (tumor necrosis factor α (TNF-α), catalog no. 88-7324, and monocyte chemotactic protein 1 (MCP-1), catalog no. 88-7391, eBioscience, San Diego, CA, USA; osteopontin, Promega, Madison, WI, USA) following the manufacturer’s instructions. Briefly, 25 mg of pooled hindlimb muscle tissues were lysed in 10 vol. of lysis buffer containing protease and phosphatase inhibitor cocktails. The lysates were incubated with the capture antibody for 3 hours at room temperature. The wells were washed five times for 3 minutes each time and incubated with detection antibodies for 2 hours at room temperature. The wells were washed as described above and incubated with avidin–horseradish peroxidase conjugates. The washing step was repeated again as above and incubated with the substrate for 15 minutes. The reactions were stopped subsequently and the absorbance was recorded at 450 nm.

### Statistics

Statistical analyses were performed using GraphPad Prism 6 software (GraphPad Software, La Jolla, CA, USA) and included two-way analysis of variance (ANOVA) followed by Tukey’s multiple comparison analysis as appropriate, as well as unpaired two-tailed *t*-tests. Data are presented as mean ± standard deviation.

## Results

### Laminin α_2_-deficient mice failed to develop in both body and muscle weight starting at week 3

**Figure 1:**
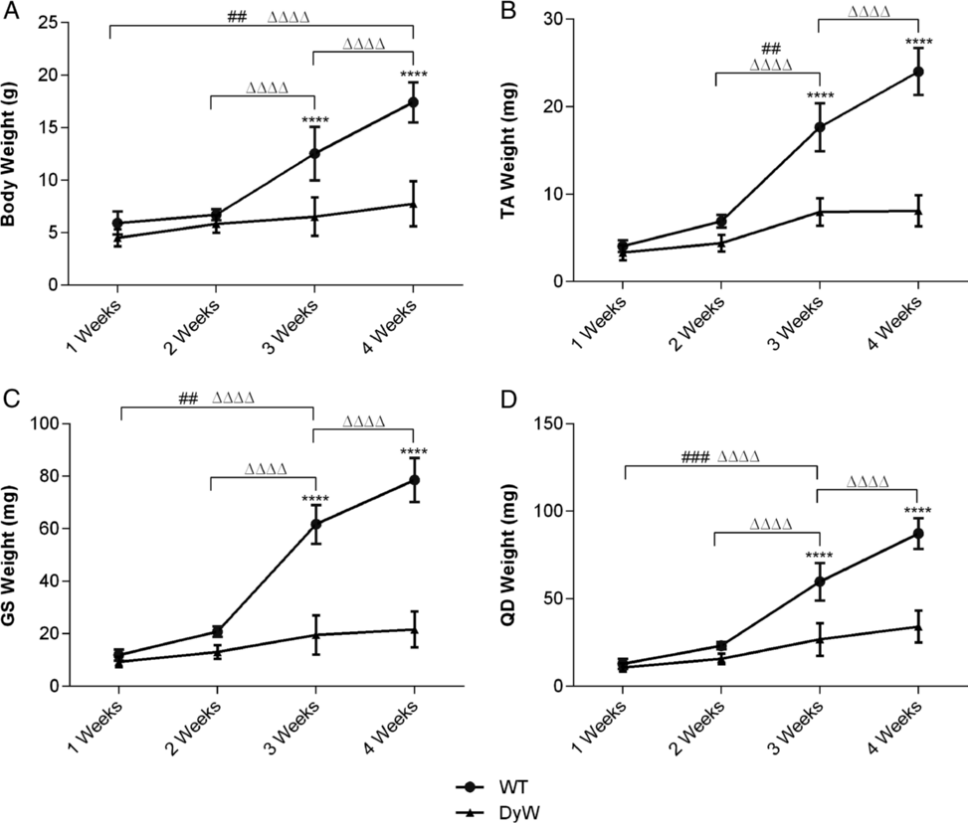
**Overall body and isolated hindlimb muscle weights show limited growth in *DyW *mice during postnatal development. (A)** At postnatal weeks 1 and 2, *Lama2*^*DyW*^ (*DyW*) mice had body weights to comparable those of wild-type (*WT*) mice, but were significantly smaller starting at week 3 (*P* < 0.05 by *t*-test (*n* = 5 to 10). **(B)** Tibialis anterior (TA), **(C)** gastrocnemius/soleus (GS) and **(D)** quadriceps (QD) muscles of *DyW* mice were also significantly smaller in weight compared to *WT* mice starting from 3 weeks of age (*P* < 0.0005 by analysis of variance (*n* = 6 to 10)). Asterisks indicate statistical significance between age-matched *DyW* and *WT* animals; hash tags indicate statistically significant changes within the *DyW* group; Delta indicates statistically significant changes within the *WT* group. *P < 0.05, **P < 0.01, ***P < 0.001, ****P < 0.0001. These statistical representations also apply to the other symbols (Δ, #).

We began our characterization of this animal model by tracking overall body and hindlimb muscle weights throughout early postnatal development. *DyW* and *WT* mice had similar body weights at 1 week (*DyW*: 4.51 ± 0.8 g, *n* = 8; *WT*: 5.92 ± 1.10 g, *n* = 5) and 2 weeks (*DyW*: 5.83 ± 0.821, *n* = 9; *WT*: 6.72 ± 0.52 g, *n* = 6) after birth (Figure 1A). *WT* mice then underwent a substantial growth spurt between weeks 2 and 4 (3 weeks: 12.54 ± 2.54 g; 4 weeks: 17.43 ± 1.91 g; *P* < 0.001 by two-way ANOVA (*n* = 6 to 10)), whereas *DyW* mice failed to substantially grow (3 weeks: 6.53 ± 1.83 g, 4 weeks: 7.76 ± 2.15 g). It should be noted that other research groups saw statistical differences in body size at postnatal day 7, though these differences were still small [17].

We next determined whether this lack of early growth correlates directly with muscle mass. Weights from the TA, GS and QD muscles followed a pattern similar to that of overall body weight (Figures 1B through 1D). Between 2 and 4 weeks, the hindlimb muscles from the *WT* mice underwent a substantial growth spurt (at 2 weeks, TA: 6.92 ± 0.70 mg, GS: 20.8 ± 1.99 mg and QD: 23.2 ± 1.99 mg; at 3 weeks, TA: 17.65 ± 2.75 mg, GS: 61.7 ± 7.28 mg and QD: 59.76 ± 10.78 mg; at 4 weeks, TA: 24.025 ± 2.65 mg, GS: 78.53 ± 8.39 mg, QD: 87.27 ± 8.74 mg; *P* < 0.0001 by two-way ANOVA (*n* = 5 to 10)), which is greatly dampened in *DyW* mice. The TA muscles of *DyW* animals showed a significant increase between weeks 2 and 3 (4.32 ± 0.96 mg to 7.98 ± 1.57 mg; *P* < 0.01 (n = 8 to 9)), whereas the GS and QD muscles showed a significant weight gain between weeks 1 and 3 (GS: 13.13 ± 2.61 mg to 21.66 ± 6.81 mg, QD: 15.76 ± 2.99 mg to 34.1 ± 9.22 mg). Even though the *DyW* hindlimb muscles underwent a small growth spurt, they did not achieve nearly the same level of growth.

### *DyW* muscles began to show histopathology as early as postnatal day 1

**Figure 2:**
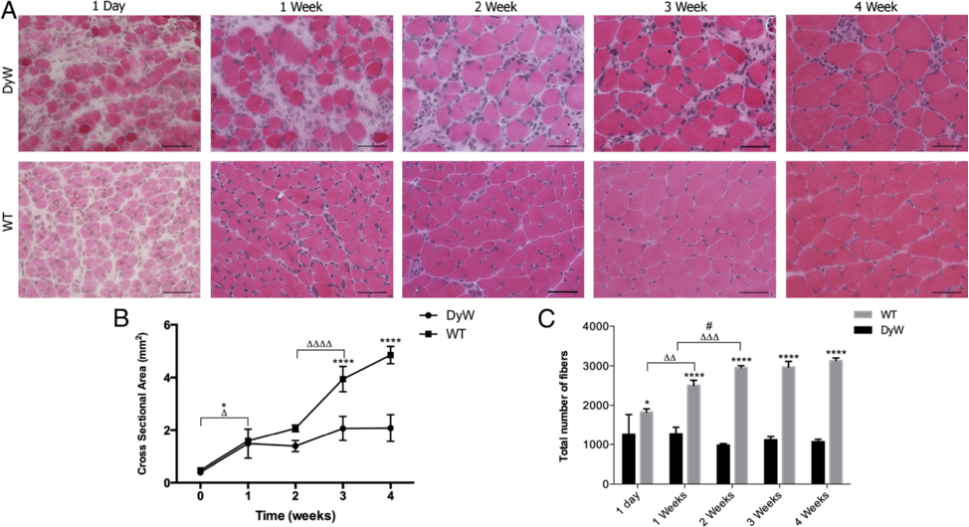
**Qualitative and quantitative analysis of hematoxylin and eosin–stained tibialis anterior muscle sections show pathology as early as 1 week of age. (A)** Qualitative histological analyses of tibialis anterior (TA) muscles show pathogenesis beginning as early as 1 day of age in *Lama2*^*DyW*^ (*DyW*) mice. These muscles have large interstitial spaces filled with infiltrating mononucleated cells. Additionally, the muscles tend to lack both consistent fiber sizes and a hierarchical organization of connective tissue. **(B)** Cross-sectional area measurements show limited growth in *DyW* mice compared to wild-type (*WT*) mice during the first 4 weeks of life. *WT* mice have significantly larger muscle cross-sections at weeks 3 and 4 compared to *DyW* mice (*P* < 0.0001 by two-way analysis of variance (ANOVA) (*n* = 3 to 5)). **(C)** Counts of the total number of muscle fibers indicate that, as early as postnatal day 1, *DyW* mice already had a decreased number of muscle cells and that this number increased during development. At all time points, *DyW* mice had significantly fewer myofibers than their *WT* counterparts (*P* < 0.0001 by two-way ANOVA (*n* = 3 to 5). Note that, though the cross-sectional area does not indicate a diference at week 1, pathology is reflected in the fiber count. Asterisks indicate statistical significance between age-matched *DyW* and *WT* animals, hash tags indicate significant changes within the *DyW* group and Deltas indicate significant changes within the *WT* group. *P < 0.05, **P < 0.01, ***P < 0.001 and ****P < 0.0001. These statistical representations also apply other symbols (Δ, #). Bar = 50 μm.

We next used hematoxylin and eosin staining of frozen TA muscle sections to look at the disease progression on the cellular level. As early as postnatal day 1, *DyW* mice exhibited obvious pathological symptoms marked by large interstitial spaces filled with infiltrating mononucleated cells, inconsistently sized myofibers and a lack of delineation of fascicles (Figure 2A). As these mice aged, their muscle fibers hypertrophied to a certain extent and occupied more of the total muscle area. However, large interstitial spaces and inconsistent fiber sizes persisted through week 4.

Interestingly, even though dystrophic mice showed obvious signs of muscle pathology at day 1, there was no discernible difference in the cross-sectional area (CSA) of their TA muscles compared to the *WT* mice (*DyW*: 0.393 ± 0.061 mm^2^, *WT*: 0.472 ± 0.076 mm^2^) (Figure 2B). However, beginning at postnatal week 3, *DyW* muscles had significantly smaller muscle cross-sections compared to age-matched *WT* muscles (3 weeks: *DyW*: 2.06 ± 0.46 mm^2^, *WT*: 3.94 ± 0.48 mm^2^; 4 weeks: *DyW*: 2.08 ± 0.51 mm^2^, *WT*: 4.86 ± 0.33 mm^2^; *P* < 0.0001 by two-way ANOVA (*n* = 3 to 5)). This follows a temporal trend similar to observed in the muscle and body weights, with *WT* mice showing a large growth spurt between weeks 2 and 3 (*P* < 0.0001 by two-way ANOVA (n = 3-4)) and *DyW* muscles failing to show much growth.

Interestingly, even though CSA and muscle weights were comparable between *DyW* and *WT* mice until postnatal week 2, dystrophic TA muscles contained roughly half the number of matured myofibers as early as week 1 (*DyW*: 1,265.25 ± 199.04, *WT*: 2,504 ± 129.64; *P* < 0.0001 by two-way ANOVA (*n* = 3 to 5)) (Figure 2C). *DyW* muscles had a small but significant decrease in total fiber number between weeks 1 and 2 (*P* < 0.05 by two-way ANOVA (*n* = 4 to 5)), whereas *WT* mice showed a significant increase in the number of myofibers during the same time frame (*P* = 0.001 by two-way ANOVA (*n* = 3 or 4). Neither group showed a significant change in fiber number after week 2 (3 weeks: *DyW*: 1,118 ± 82.6, *WT*: 2,968.67 ± 144.81; 4 weeks: *DyW*: 1,069 ± 61.77, *WT*: 3,132.33 ± 68.62).

### *DyW* mice had increased incidence of apoptosis and expression of myogenic markers throughout postnatal development

**Figure 3:**
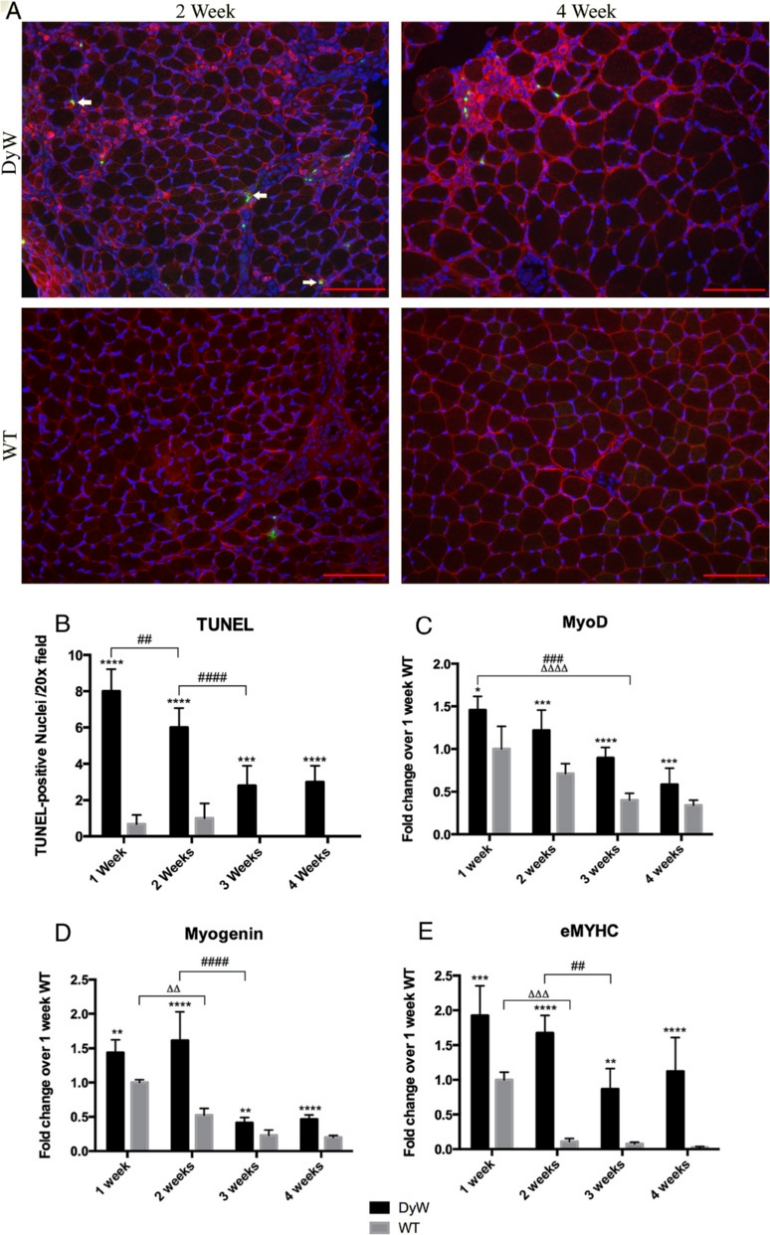
**Gene expression analysis using quantitative RT-PCR, along with TUNEL immunostaining, show an increase in the myogenic program and cell death. (A)** Terminal deoxynucleotidyl transferase 2′-deoxyuridine-5′-triphosphate nick-end labeling (TUNEL) staining shows an increased number of apoptotic nuclei in *Lama2*^*DyW*^ (*DyW*) muscles at each of the four time points. Desmin costaining was used to delineate differentiated myogenic cells. Arrows point to those cells that are both desmin-positive (red) and TUNEL-positive (green). (DyW is also referred to as KO, Knockout.) **(B)** Quantitative analysis of the TUNEL staining shows the highest incidence of apoptosis at postnatal week 1, which decreased by 50% at week 3. **(C)** Gene analysis of MyoD shows the highest expression for both groups early in life, followed by decreases over time. *DyW* mice had significantly increased expression compared to age-matched wild-type (*WT*) mice at all time points (1 week: *P* < 0.05, 2 weeks: *P* < 0.001, 3 weeks: *P* < 0.0001 and 4 weeks: *P* < 0.001, all by two-way analysis of variance (ANOVA) (*n* = 5 for all groups). **(D)** Myogenin expression shows a similar trend of having the highest expression early on, and *DyW* levels were also increased compared to all age-matched *WT* groups (1 week: *P* < 0.01, 2 weeks: *P* < 0.0001, 3 weeks: *P* < 0.005 and 4 weeks: *P* < 0.0001, all by two-way ANOVA (n = 5 for all groups). **(E)** Embryonic myosin heavy chain (eMYHC) expression was significantly elevated in the *DyW* mice at each of the four time points (1 week: *P* < 0.0005, 2 weeks: *P* < 0.0001, 3 weeks: *P* < 0.005 and 4 weeks: *P* < 0.0001, all by two-way ANOVA (*n* = 5). Asterisks indicate statistical significance between age-matched *DyW* and *WT* animals, hash tags indicate significant changes within the *DyW* group and Deltas indicate significant changes within the *WT* group. *P < 0.05, **P < 0.01, ***P < 0.001 and ****P < 0.0001. These statistical representations also apply other symbols (Δ, #). Bar = 100 μm.

TUNEL staining of TA muscle sections showed that there was an elevated number of apoptotic nuclei in *DyW* animals throughout the early development time points. Representative images of 2- and 4-week-old animals are shown in Figure 3A. We demonstrate that both mature muscle fibers and cells within the interstitium undergo apoptosis in these tissues. Importantly, we observed that at earlier time points, a subset of those TUNEL-positive nuclei in the interstitial space are also desmin-positive (one of the earliest markers for differentiated myogenic cells). This is in contrast to older tissues, where TUNEL-positive nuclei were mostly limited to the mature myofibers and desmin-negative interstitial cells. Quantitation of TUNEL-positive nuclei shows that apoptosis was significantly increased in the *DyW* mice compared to the *WT* mice at all time points (*P* < 0.001 by two-way ANOVA (*n* = 3)) and also that it was most abundant in *DyW* mice at weeks 1 and 2 and became less prevalent by weeks 3 and 4 (*P* < 0.0001 by two-way ANOVA (*n* = 3)) (Figure 3B). We observed few TUNEL-positive nuclei in the *WT* mice in the first 2 weeks and virtually none at subsequent time points.

To determine whether *DyW* muscles attempt to compensate for lost muscle cells through upregulation of the myogenic program, we looked at the gene expression of MyoD, myogenin and embryonic myosin heavy chain (eMYHC, myosin heavy chain 3 (*MYH3*)) as markers of myogenesis. In these gene expression studies, we used 1-week *WT* values for normalization throughout postnatal development. Comparing the expression of all groups to the *WT* at a single time point allowed us to observe changes in both healthy and dystrophic animals over time, which told us not only whether *DyW* mice had dysregulated gene expression compared to age-matched *WT*, but also how they vary from normal developmental progression. See Additional file 1: Table S1 for data concerning *DyW* expression levels relative to age-matched *WT* mice.

*WT* mice expressed their highest levels of MyoD, myogenin and MYH3 at postnatal week 1, which then decreased with age. MyoD expression was significantly lower in both groups by week 3 compared to week 1 (*P* < 0.0001 for *WT* and *P* < 0.001 for *DyW* by two-way ANOVA (*n* = 5 for all groups)) (Figure 3B). Myogenin showed a similar but sharper decrease in expression between weeks 2 and 3 (*P* < 0.0001 by two-way ANOVA (*n* = 5)) (Figure 3C). At all four time points, both myogenin and MyoD were significantly upregulated in *DyW* muscle compared to age-matched *WT* muscle (Additional file 1: Table S1) (1 week: *P* < 0.05, 2 and 4 weeks: *P* < 0.001, 3 weeks: *P* < 0.0001, all by two-way ANOVA (*n* = 5 for all groups)). Expression of eMYHC in *WT* muscles was elevated at week 1, followed by a sharp decrease by week 2 (*P* < 0.001 by two-way ANOVA (*n* = 5), which remained low through week 4 (Figure 3D). In contrast, eMYHC gene expression was markedly higher in the *DyW* muscles at all time points compared to age-matched *WT* muscles (*P* < 0.0005 at week 1, *P* < 0.0001 at weeks 2 and 4 and *P* < 0.005 at week 3, all by two-way ANOVA (*n* = 5)) (Additional file 1: Table S1). It is important to note that though this upregulation was only twofold at week 1, this increase was over thirty-fold by week 4.

### Persistent macrophage infiltration was observed as early as postnatal week 1 in *DyW* muscle

**Figure 4:**
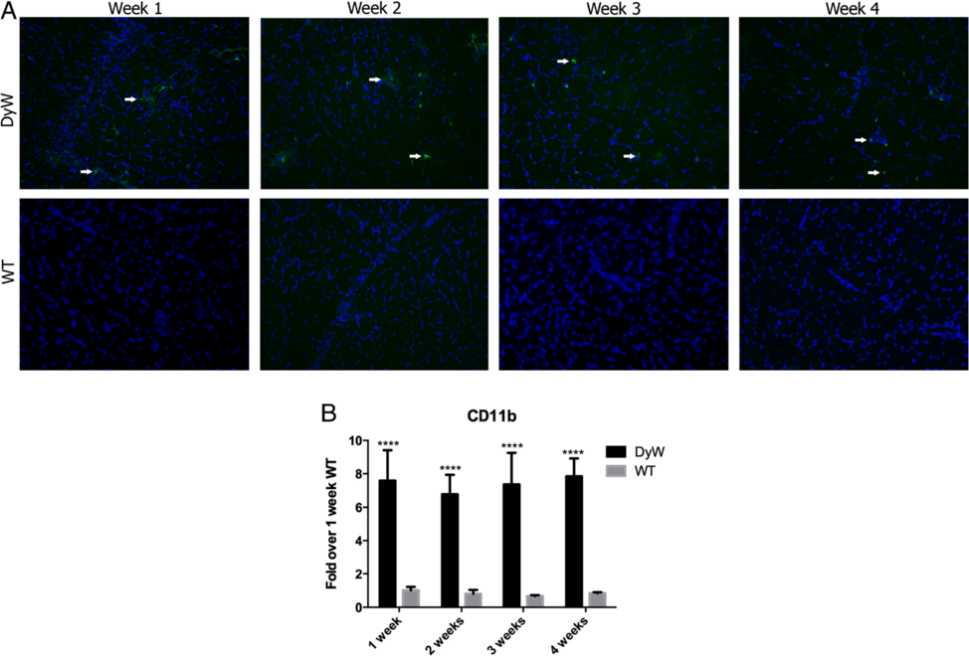
**Mac-1 staining and quantitative RT-PCR of CD11b show elevated macrophage infiltration throughout postnatal development. (A)** Mac-1 immunostaining of tibialis anterior muscle sections shows high macrophage infiltration as early as postnatal week 1 in *Lama2*^*DyW*^ (*DyW*) mice. Wild-type (*WT*) mice had minimal macrophage presence at weeks 1 and 2 and virtually none by weeks 3 and 4. **(B)** Confirmation of these results by CD11b quantitative RT-PCR shows consistent seven- to elevenfold increases in expression compared to age-matched *WT* (*P* < 0.0001 by two-way analysis of variance for all groups (n = 5 for all groups)). Asterisks indicate statistical significance between age-matched *DyW* and *WT* animals, hash tags indicate significant changes within the *DyW* group and Delta indicate significant changes within the *WT* group. *P < 0.05, **P < 0.01, ***P < 0.001 and ****P < 0.0001. These statistical representations also apply other symbols (Δ, #). Bar =100 μm.

Immunostaining of TA muscle sections with Mac-1 antibody (CD11b, a marker for macrophages) revealed that *WT* muscles had a limited number of macrophages at week 1, a sparse presence by week 2 and virtually none by weeks 3 and 4 (Figure 4A). Conversely, *DyW* mice had a high level of macrophage infiltration as early as 1 week of age, which continued throughout postnatal development. Quantitative RT-PCR analysis confirmed these results by showing increased expression of CD11b at all time points (*P* < 0.0001 by two-way ANOVA (*n* = 5)) (Figure 4B).

### *DyW* muscles showed a dynamic inflammatory environment beginning at week 1

**Figure 5:**
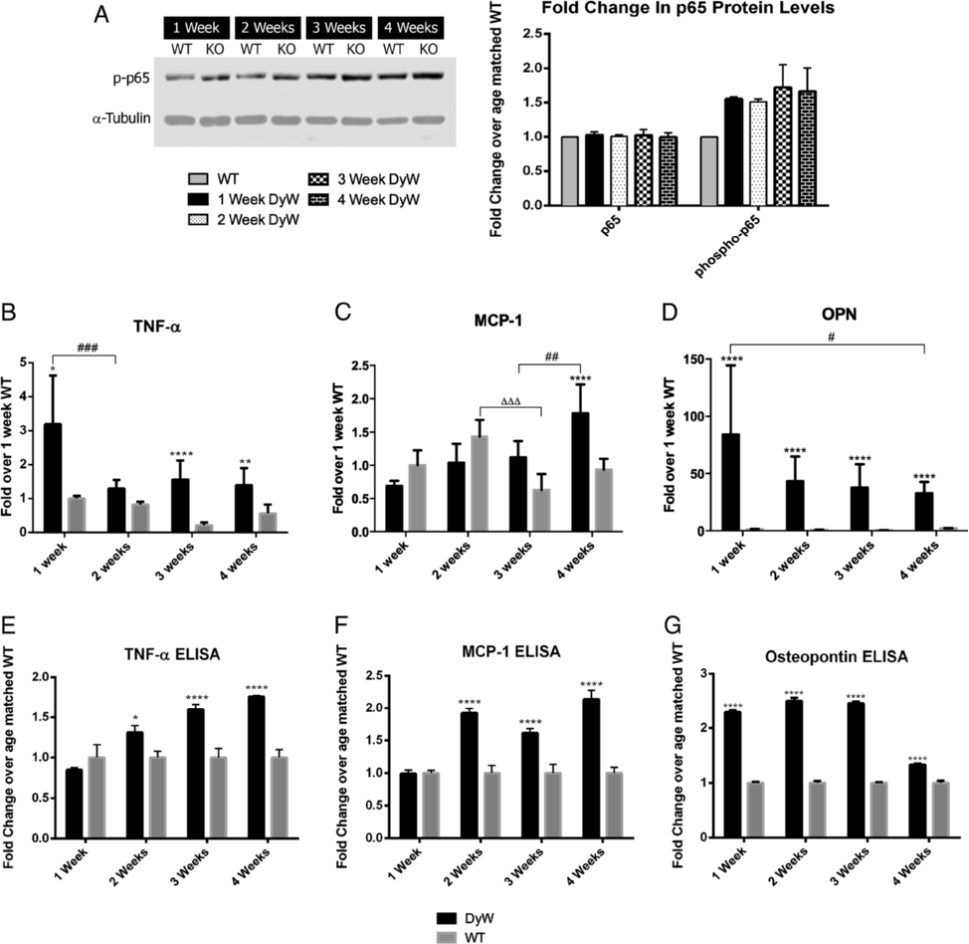
**Western blot analysis, qRT-PCR and ELISA show increased levels of NFκB–mediated inflammation starting at postnatal week 1 and continuing through development. (A)** Western blot of total and phosphorylated p65 reveals that *DyW* mice had elevated levels of NF-κB signaling beginning at postnatal week 1 and continuing throughout development. **(B)** TNF-α gene analysis shows that DyW muscles had significantly elevated expression at week 1 (*P *< 0.01), week 3 (*P* < 0.0001) and week 4 (*P *< 0.05 by two-way ANOVA (*n* = 5) for all groups). **(C)** Gene expression analysis of MCP-1, reveals that *DyW* and WT mice had comparable expression levels until postnatal week 4, at which point the *DyW* MCP-1 level became significantly elevated (*P* < 0.0005 by two-way ANOVA (*n* = 5)). **(D)** Osteopontin (OPN) was also highly upregulated (>25 –fold) at every time point (*P* < 0.0005 by two-way ANVOA (*n* = 5) for all groups). **(E)** ELISA of TNF-α shows elevated expression levels in *DyW* mice starting at week 2 (*P* < 0.05 for week 2, *P *< 0.0001 for weeks 3 and 4, both by two-way ANOVA (*n *= 3)). **(F)** MCP-1 was also found to be upregulated at weeks 2, 3 and 4 (*P* < 0.0001 by two-way ANOVA (*n* = 3) for all groups). **(G)** Osteopontin protein was also seen to be upregulated at each of the four time points (*P* < 0.0001 by two-way ANOVA (*n* = 5)), with the largest increases seen in the first 3 weeks. Asterisks indicate statistical significance between age-matched *DyW* and WT, hash tags indicate significant changes within the *DyW* group and Deltas indicate significant changes within the WT group. *P < 0.05, **P < 0.01, ***P < 0.001 and ****P< 0.0001. These statistical representations also apply other symbols (Δ, #).

We have previously shown that nuclear factor κB (NF-κB), and, more specifically, the activation of its p65 subunit, is upregulated at week 8 in *DyW* animals [19]. To see if this dysregulation is evident at earlier time points, we performed Western blot analysis of p65 and phospho-p65. We saw elevated levels of phospho-p65 (1.4-fold) as early as postnatal week 1, though we observed no change in total p65 (Figure 5A, Full blot can be found in Additional file 2).

To further explore the extent of inflammation in these mice, we looked at the expression of various inflammatory genes, beginning with the NF-κB-related cytokine TNF-α (Figure 5B). In *WT* development, there was no observed variation in expression between postnatal weeks 1 and 4. Within the *DyW* group, there was a significant decrease in TNF-α expression between weeks 1 and 2 (*P* < 0.001 by two-way ANOVA (*n* = 5), followed by no significant expression changes through week 4. However, *DyW* mice exhibited significantly elevated TNF-α expression at week 1 (*P* < 0.01), week 3 (*P* < 0.0001) and week 4 (*P* < 0.05, all by two-way ANOVA) (*n* = 5) compared to age-matched *WT*. Interestingly, MCP-1, another NF-κB-related cytokine, became upregulated only at postnatal week 4 (*P* < 0.0001 by two-way ANOVA (*n* = 5)) (Figure 5C). Osteopontin (a gene associated with the inflammatory environment of dystrophic mice which has been shown to have a correlation with muscle scarring) showed massive upregulation in *DyW* mice at all time points (*P* < 0.0001 by two-way ANOVA (*n* = 5)) (Figure 5D). *DyW* animals had a nearly 100-fold increase in osteopontin expression over the *WT* mice at week 1, which significantly decreased by week 4 (*P* < 0.05 by two-way ANOVA (*n* = 5)). *WT* mice had no significant changes in osteopontin expression between any time points.

ELISAs also revealed upregulation of these inflammatory markers on the protein level. Although we saw the greatest upregulation of TNF-α gene expression at postnatal week 1, we actually did not see any change in protein levels compared to the *WT* mice at this time point (Figure 5E). However, we did find protein expression of TNF-α to be increased between 1.3- and 1.7-fold in *DyW* animals throughout the rest of early postnatal development (2 weeks: *P* < 0.05, and 3 and 4 weeks: *P* < 0.0001, both by two-way ANOVA (*n* = 5)). Additionally, though we did not see an increase in MCP-1 mRNA before postnatal week 4, we saw a 1.9-fold upregulation in the protein as early as week 2, which then remained elevated through week 4 (*P* < 0001 by two-way ANOVA (*n* = 5) for all time points) (Figure 5F). Similar to what we saw in gene expression, osteopontin protein was upregulated at all time points, with the greatest increases seen at weeks 1, 2 and 3 (*P* < 0.0001 by two-way ANOVA (*n* = 5) for all time points) (Figure 5G).

### Moderate increase in collagen levels was observed in early postnatal *DyW* development

**Figure 6:**
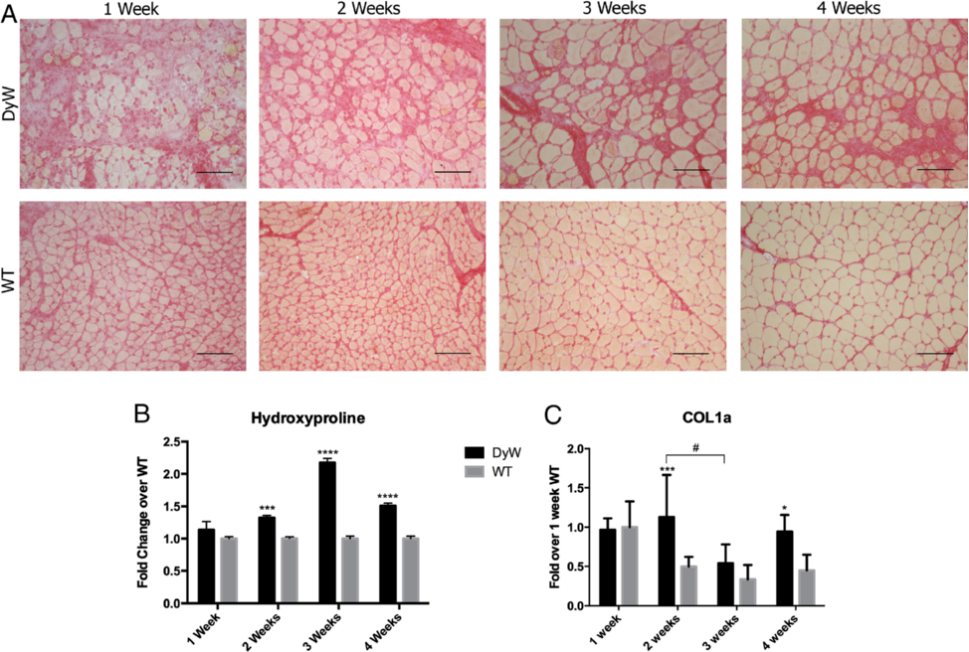
**Picro-Sirius Red staining, hydroxyproline assay and quantitative RT-PCR show moderately elevated levels of collagen in *DyW *muscle. (A)** Qualitative analysis of Picro-Sirius Red staining done on frozen muscle sections from *Lama2*^*DyW*^ (*DyW*) and wild-type (*WT*) animals during the first 4 weeks after birth shows increased presence of collagen by weeks 3 and 4. **(B)** Hydroxyproline assay shows significantly elevated levels of collagen at weeks 2, 3 and 4 (*P* = 0.0001 at week 2, *P* < 0.0001 at weeks 3 and 4, all by two-way analysis of variance (ANOVA) (*n* = 3)). **(C)** Quantitative RT-PCR shows similar *Col1a* (collagen, type I, α1) transcript levels between the two groups at weeks 1 and 3, with *DyW* mice having significantly increased expression at weeks 2 and 4 (*P* < 0.05 for week 4 and *P* < 0.001 for week 2, both by two-way ANOVA (*n* = 5 for all groups). It should be noted that collagen gene expression was extremely variable in both the *DyW* and *WT* animals at all time points. Asterisks indicate statistical significance between age-matched *DyW* and *WT* animals, hash tags indicate significant changes within the *DyW* group and Deltas indicate significant changes within the *WT* group. *P < 0.05, **P < 0.01, ***P < 0.001 and ****P < 0.0001. These statistical representations also apply other symbols (Δ, #). Bar = 100 μm.

Picro-Sirius Red staining showed increased fibrotic areas in *DyW* mice by week 3 (Figure 6A). At postnatal week 1, Picro-Sirius Red staining in both *DyW* and *WT* mice was fairly faint, although *DyW* mice had much greater fibrotic area in their muscles. To confer further specificity to Sirius red staining [22], we viewed sections under polarized light (a method of viewing Picro-Sirius Red–stained thick collagen fibers). Neither *DyW* nor *WT* muscles showed a high presence of collagen fibers at week 1 (data not shown). In contrast, by week 3, *WT* muscles exhibited minimal interstitial spaces, whereas age-matched *DyW* muscles still has large spaces between fibers that now stained a deep red. Again, these slides were viewed under polarized light, which revealed limited amounts of collagen deposition in the *WT* muscles, even in those areas stained red, and a much higher presence in the *DyW* muscles (data not shown). We then quantified our results by performing a hydroxyproline assay (Figure 6B) and found no difference between *DyW* and *WT* hydroxyproline levels at week 1. By week 2, we saw a 1.3-fold upregulation (*P* = 0.0001 by *t*-test (*n* = 3)), which was followed by a 2.2-fold increase at week 3 (*P* < 0.0001 by *t*-test (*n* = 3)), and a 1.5-fold increase at week 4 (P < 0.0001 by *t*-test (n = 3)). Next, we looked at the gene expression of *Col1a* (collagen, type I, α1) at these four time points (Figure 6C). We saw no change in transcript levels at week 1, but we saw a significant elevation in mRNA expression levels compared to age-matched WT at 2 weeks (*P* < 0.001) and 4 weeks (*P* < 0.05, both by two-way ANOVA) (*n* = 5). Interestingly, the largest increase in expression was at postnatal week 2, before we saw any major increase with hydroxyproline or Picro-Sirius Red.

### qRT-PCR and Western blots demonstrate dysregulation of TGF-β-mediated fibrosis and ECM remodeling

**Figure 7:**
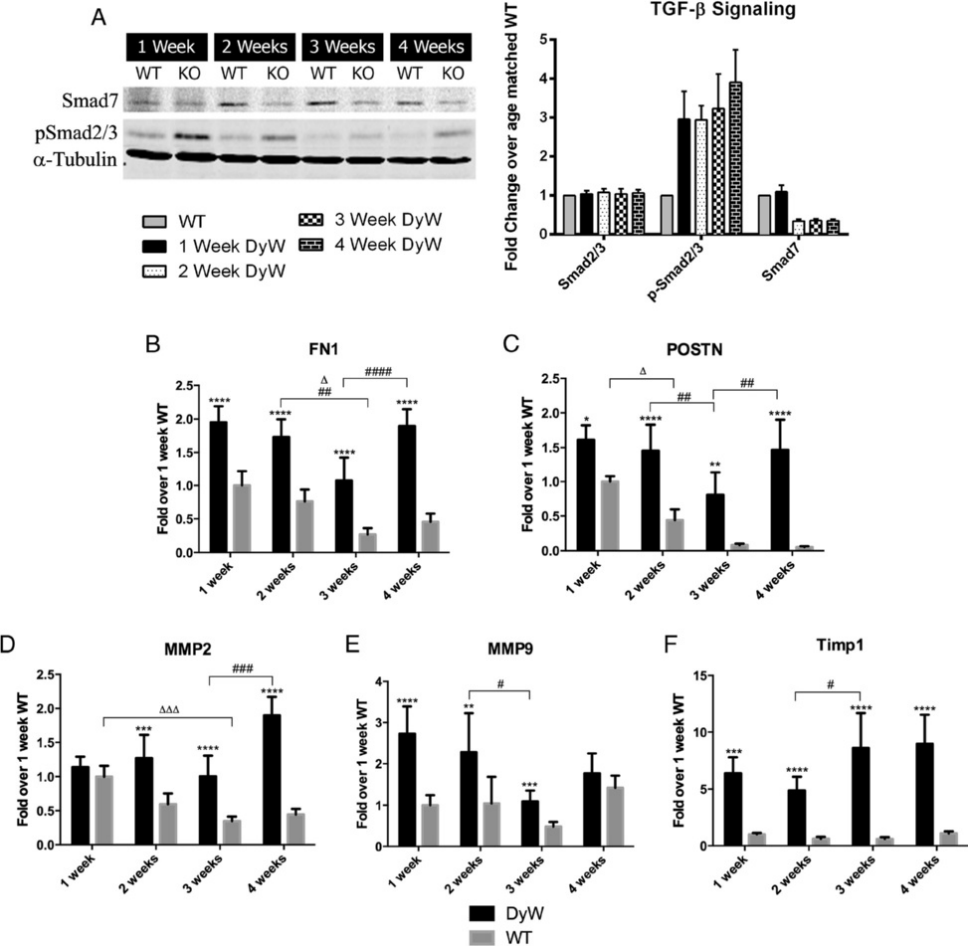
**Western blot and quantitative RT-PCR show upregulated extracellular matrix remodeling pathways in *DyW *mice throughout postnatal development. (A)** Western blot of total and phosphorylated Smad2/3 (pSmad2/3) shows an increase in transforming growth factor β (TGF-β) signaling as early as week 1. Additionally, we saw a decrease in Smad7 expression as early as week 2. (DyW is also referred to as KO, Knockout.) **(B)** Fibronectin 1 (*Fn1*) gene expression was found to be upregulated two- to fivefold in *Lama2*^*DyW*^ (*DyW*) animals at all four time points compared to age-matched wild-type (*WT*) mice (*P* < 0.0001 by two-way analysis of variance (ANOVA) and *n* = 5 for all groups). **(C)** Additionally, periostin (*Postn*) transcript levels showed massive upregulation at weeks 2, 3 and 4 (*P* < 0.05 for week 1, *P* < 0.005 for week 3 and *P* < 0.0001 for weeks 2 and 4, all by two-way ANOVA (*n* = 5 for all groups)). **(D)** Matrix metalloproteinase 2 (MMP-2) transcription levels became elevated by postnatal week 2 and remained that way through week 4 (*P* < 0.0005 by two-way ANOVA (*n* = 5)). **(E)** In contrast, MMP-9 expression was significantly upregulated at weeks 1, 2 and 3 (*P* < 0.005 by two-way ANOVA (*n* = 5)), but was comparable to the *WT* by week 4. **(F)** Gene expression of tissue inhibitor of matrix metalloproteinase 1 (TIMP-1) shows a several-fold upregulation starting as early as week 1 (*P* < 0.0005 by two-way ANOVA (*n* = 5)). Asterisks indicate statistical significance between age-matched *DyW* and *WT* animals, hash tags indicate significant changes within the *DyW* group and Deltas indicate significant changes within the *WT* group. *P < 0.05, **P < 0.01, ***P < 0.001 and ****P < 0.0001 These statistical representations also apply other symbols (Δ, #).

We next performed Western blot analysis of Smad2/3, a transcription factor downstream of TGF-β that induces the expression of collagen and other fibrotic proteins. Although Western blot analysis showed no increase in total Smad2/3 until week 2, there was a threefold increase in the activated (phosphorylated) form as early as week 1 (Figure 7A, Full blot can be found in Additional file 2). Throughout the rest of development, both total Smad2/3 and phospho-Smad2/3 were upregulated in *DyW* animals. We also looked at the expression of Smad7, a protein that inhibits TGF-β signaling at the level of the receptor as well as in the nucleus [23,24]. We found that, in addition to Smad2/3 being upregulated, Smad7 expression was decreased at weeks 2 through 4.

We also examined the expression of two other fibrosis-related genes downstream of TGF-β: fibronectin (*Fn1*) and periostin (*Postn*). *WT* animals have their highest *Fn1* expression in the first 2 weeks after birth, which is followed by a significant decrease between weeks 2 and 3 (*P* < 0.05 by two-way ANOVA (*n* = 5)) (Figure 7B). In contrast, though *Fn1* expression in *DyW* muscles also decreased between weeks 2 and 3 (*P* < 0.01 by two-way ANOVA (*n* = 5)), it increased significantly between weeks 3 and 4 (*P* < 0.0001 by two-way ANOVA (*n* = 5)). At each of these early time points, *Fn1* expression showed a two- to fourfold upregulation in *DyW* animals compared to age-matched *WT* (*P* < 0.0001 by two-way ANOVA (*n* = 5)). *Postn* was also upregulated in *DyW* animals throughout most of development (Figure 7C). *WT* mice had their highest levels of *Postn* expression at postnatal week 1, which significantly decreased by week 3 (*P* < 0.0001 by two-way ANOVA (*n* = 5)). Like *Fn1* expression, expression of *Postn* in *DyW* muscles showed a decrease between weeks 2 and 3 (*P* < 0.01 by two-way ANOVA (*n* = 5), followed by an upregulation between weeks 3 and 4 (*P* < 0.01 by two-way ANOVA (*n* = 5)). At all time points, *DyW* mice had significantly increased expression in *POSTN* compared to age-matched *WT* mice (*P* < 0.05 at week 1, *P* < 0.0001 at weeks 2 and 4 and *P* < 0.005 at week 3, all by two-way ANOVA (*n* = 5)).

We found that the gene expression of matrix metalloproteinases (MMPs) 2 and 9, proteins that participate in the degradation of collagen and other (ECM) components [24,(25], were upregulated in *DyW* muscles throughout development. Expression of MMP-2 in *WT* muscles began high and decreased with age, becoming significantly lower by week 3 (*P* < 0.001 by two-way ANOVA (*n* = 5)) (Figure 7D), whereas MMP-9 showed no fluctuation over time (Figure 7E). In *DyW* mice, MMP-2 expression was consistent at weeks 1 through 3 and increased significantly by week 4 (*P* < 0.0001 by two-way ANOVA (*n* = 5). MMP-9 expression, on the other hand, was at its highest early on and decreased between weeks 2 and 3 (*P* < 0.05 by two-way ANOVA (*n* = 5). In comparison to age-matched *WT* mice, in *DyW* mice, MMP-2 was found to be significantly upregulated at weeks 2, 3 and 4 (*P* < 0.0005 by two-way ANOVA (*n* = 5)), and MMP-9 was found to be overexpressed at weeks 1, 2 and 3 (*P* < 0.005 by two-way ANOVA (*n* = 5)). Interestingly, tissue inhibitor of matrix metalloproteinase 1 (TIMP-1) was also upregulated at every time point (*P* < 0.0005 by two-way ANOVA (*n* = 5)) and to a much greater degree than either MMP-2 or MMP-9 (Figure 7F). We saw no significant TIMP-1 fluctuations in *WT* animals.

### Immunohistochemistry shows increased presence of fibronectin as early as postnatal week 1

**Figure 8:**
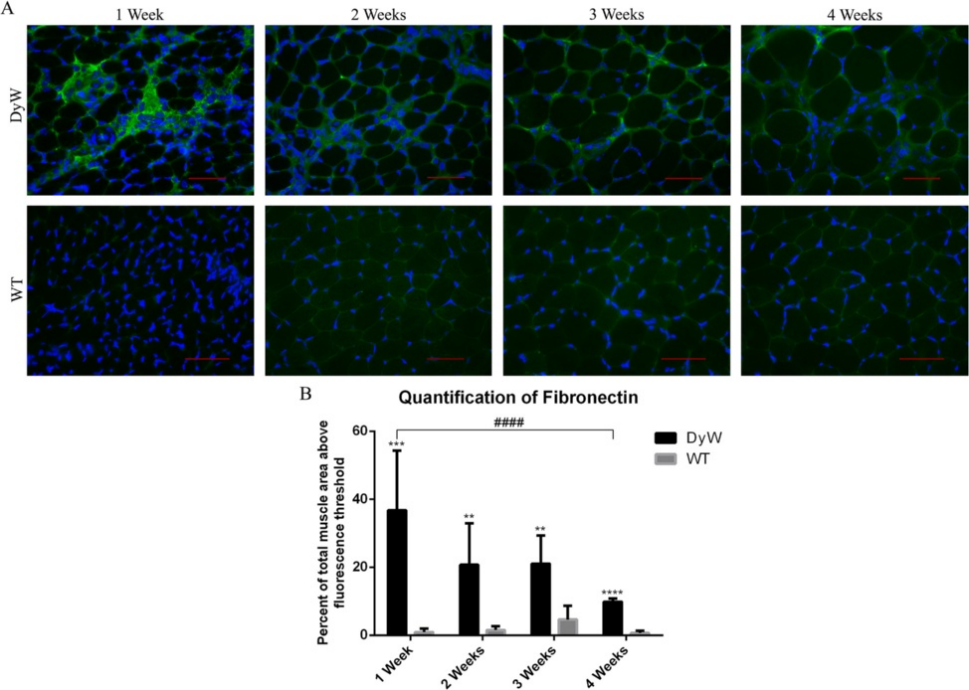
**Qualitative analysis of fibronectin 1–stained frozen tibialis anterior muscle sections from *DyW *and *WT *mice at postnatal weeks 1 through 4. (A)** Qualitative analysis shows that the highest levels of fibronectin in the extracellular matrix of *Lama2*^*DyW*^ (*DyW*) mice appear to have occurred at postnatal week 1. WT, Wild type. **(B)** Quantification using fluorescence thresholding confirms the immunohistochemical results and shows that *DyW* mice have elevated fibronectin expression at each time point (*P* < 0.01 by *t*-test (*n* = 3)). Though this expression decreased with age, fibronectin remained persistently upregulated throughout postnatal development. Bar = 50 μm.

Immunostaining with fibronectin antibodies revealed that, as early as postnatal week 1, there was an extremely increased presence of fibronectin in the interstitial spaces of *DyW* animals compared to the *WT* (Figure 8A). We performed quantitative analysis based on signaling intensities using a predetermined threshold. These data show that, at postnatal week 1, 40% of total *DyW* muscle area exhibited fibronectin staining above a given fluorescence threshold, compared to approximately 1% in the *WT* (*P* < 0.001 by *t*-test (*n* = 3)) (Figure 8B). The signal intensity remained higher in the *DyW* animals at each time point, though it was definitely most pronounced in the first 3 weeks after birth (*P* < 0.01 by *t*-test (*n* = 3).

### qRT-PCR shows dysregulation of angiotensin system by week 2

**Figure 9:**
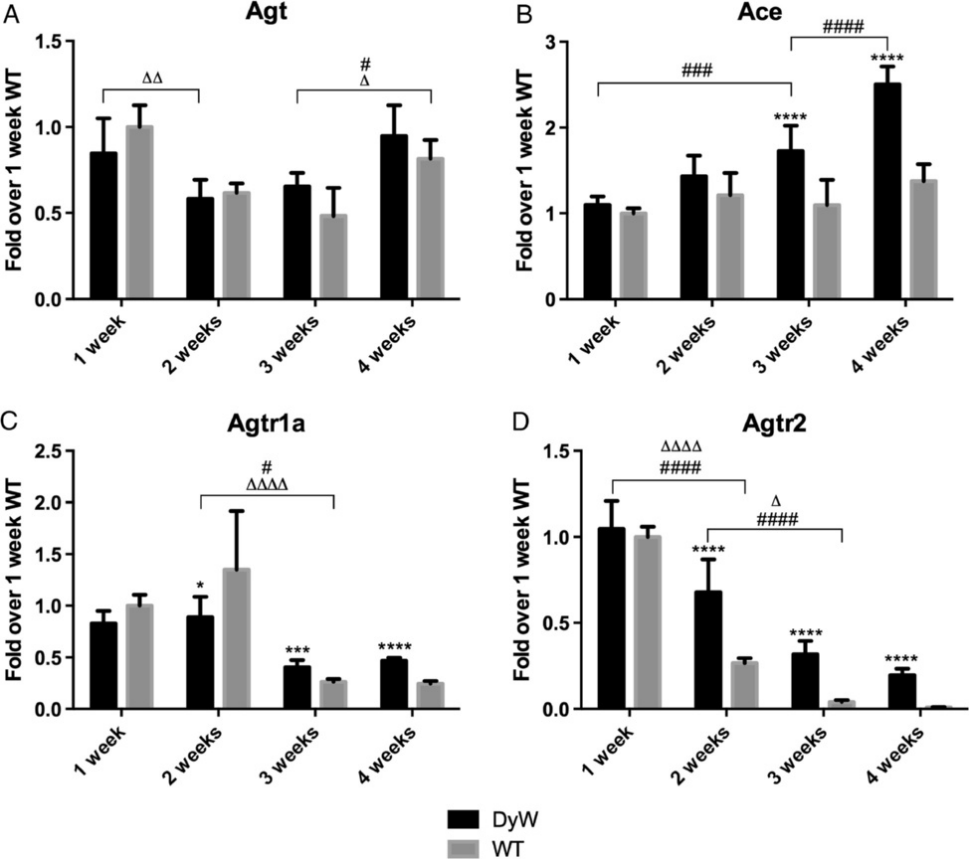
**Quantitative RT-PCR of angiotensin-related genes shows dysregulation of the renin-angiotensin signaling system in *DyW *muscle. (A)** Gene analysis of angiotensin (*Agt*) shows no difference in expression between *Lama2*^*DyW*^ (*DyW*) and wild-type (*WT*) animals from postnatal weeks 1 through 4. **(B)** We found that angiotensin-converting enzyme (*Ace*) was significantly upregulated by week 3 (*P* < 0.005, by two-way analysis of variance (ANOVA) (*n* = 5)). **(C)** Angiotensin II type 1a receptor (*Agtr1a*) was also upregulated starting at week 3 in these mice (*P* < 0.001 by two-way ANOVA (*n* = 5). **(D)** We found that the expression of angiotensin II type 2 receptor (*Agtr2*) was increased several-fold over the *WT* mice starting at week 2 (*P* < 0.0001 by two-way ANOVA (*n* = 5)). Note that, though it does not entirely come across in the graph, there is a 25-fold increase in *Agtr2* expression at week 4. Asterisks indicate statistical significance between age-matched *DyW* and *WT* animals, hash tags indicate significant changes within the *DyW* group and Deltas indicate significant changes within the *WT* group. *P < 0.05, **P < 0.01, ***P < 0.001 and ****P < 0.0001. These statistical representations also apply other symbols (Δ, #).

Overexpression of angiotensin has recently been implicated to drive both inflammation and fibrosis through the NF-κB and TGF-β pathways, respectively [25-27]. We saw that *DyW* mice had no significant differences in angiotensin expression compared to age-matched *WT* through week 4 (Figure 9A). Although *WT* levels of angiotensin-converting enzyme (*Ace*) did not fluctuate over time, *DyW* animals overexpressed *Ace* by weeks 3 and 4 (*P* < 0.0001 by two-way ANOVA (*n* = 5)) (Figure 9B). Additionally, angiotensin II receptor type 1a (Agtr1a) and type 2 (*Agtr2*) in the *WT* had high expression early in development, which then decreased with time. *DyW* mice showed a similar pattern, but failed to achieve this same decrease in expression, leading to a significant overexpression of *Agtr1a* at weeks 3 and 4 (*P* < 0.001 by two-way ANOVA (*n* = 5)) (Figure 9C) and of *Agtr2* at weeks 2, 3 and four (*P* < 0.0001 by two-way ANOVA (*n* = 5) (Figure 9D). We would like to note that, though the extent of upregulation of *Agtr2* at week 4 does not come across on the graph in Figure 9D, *DyW* mice had a 20-fold increase compared to age-matched *WT* animals.

## Discussion

Patients with MDC1A have been shown to exhibit impaired muscle growth and progressive inflammation, followed by extensive fibrosis very early in life [28,29]. We observed that *DyW* and *WT* mice have comparable body and muscle weights at week 1, only becoming drastically different by week 3, when *WT* mice began a large growth spurt that was lacking in *DyW* animals. Although an overt phenotype was not apparent at week 1, our observations at the cellular level reveal that pathology was already rampant. Our histological analysis shows that, even though muscle CSAs were comparable at week 1, only a small proportion of *DyW* muscle tissue was actually occupied by myofibers, with the rest of the muscle being taken up by large interstitial spaces that contained a high number of mononucleated cells and fibrillar proteins. In fact, as early as week 1, *DyW* muscles contained only half the number of myofibers as their *WT* counterparts.

We believe that the decreased number of myofibers present in these muscles is partly explained by either impaired myogenesis or increased muscle degeneration. We found that *DyW* mice exhibited high numbers of apoptotic nuclei throughout early development. This is in congruence with the findings in other laboratories in which researchers have shown massive muscle fiber degeneration early in the pathology of patients with MDC1A as well as in *DyW*, *dy/dy* and *Dy3K* mice [14-16,18]. Our data also show that *DyW* muscles upregulated the expression of myogenic genes, including eMYHC, likely in an attempt to compensate for this loss of fibers. Wardrop and Dominov [17] also noted an elevation of eMYHC protein in *DyW* mice, and Reggianni *et al*., who also saw this in the *dy/dy* model, argued that this is an indicator of delayed differentiation in laminin-deficient muscles [30]. Further, it has previously been demonstrated that partially differentiated myofibers are prone to apoptosis [31], and it has been suggested that the lack of regeneration seen in laminin-deficient mice is actually due to the death of newly formed myofibers [32,33]. In line with these hypotheses, we saw the largest presence of apoptotic nuclei in *DyW* mice at postnatal week 1, the time point when these mice would have the largest number of newly formed fibers. In fact, at earlier time points we saw many small desmin-positive cells in the interstitium undergoing apoptosis, which was less prevalent at later time points. Our results suggest that, though antiapoptotic therapies could be useful throughout the lives of these mice, they may have the greatest efficacy very early in postnatal development by helping to preserve the population of developing myofibers. We have demonstrated that genetic inhibition of apoptosis by mutation of the *BAX* gene in these mice results in the most robust outcome of all apoptotic therapies that have been tried in this disease [34,35].

We believe that, because of the extracellular origins of this disease, abnormalities in the myomatrix [36] and extracellular environment of these mice are paramount to the progression of *DyW* pathology. Our unpublished results and the work of investigators in other laboratories actually show that laminin-deficient muscles have dysregulation of matricellular proteins, molecules that are structurally unrelated components of the extracellular matrix that, under normal circumstances, are elevated only in development or in response to injury [13,17]. These matricellular proteins, such as tenascin C and thrombospondins, along with various cytokines, growth factors and cell surface receptors, play a major role in signaling and tissue remodeling in pathological scenarios [37,38]. In this study, we provide a comprehensive set of novel results focused on the expression of a few such molecules in *DyW* mice, including osteopontin, periostin, and fibronectin, that may help to explain the link between inflammation and pathological tissue remodeling in this disease.

It has been established that chronic inflammation inhibits muscle regeneration, promotes apoptosis and leads to the dysregulation of matricellular proteins [39-42]. Indeed, researchers at various laboratories have shown that inflammation is one of the earliest pathologies seen in many dystrophies, including in patients with MDC1A [18,43]. Here we show that laminin-deficient muscles have a high infiltration of macrophages from a very early age, which remains a persistent issue throughout development. These findings are coincident with increased NF-κB signaling and upregulation of NF-κB related cytokines, all of which are known to promote inflammation, inhibit myogenesis and increase protein degradation [42,44,45]. What is particularly interesting about our findings is the massive, persistent transcriptional dysregulation of osteopontin in the first 4 weeks after birth. Osteopontin is an adhesive ECM protein that also plays a large role in integrin-mediated cell signaling and can be secreted by both regenerating muscle and macrophages [46,47]. It has been shown that certain base levels of osteopontin can promote muscle regeneration and recruit macrophages in response to injury, but that pathologically elevated levels of osteopontin inhibit myogenesis and promote chronic inflammation and TGF-β-mediated fibrosis [47-50]. Therefore, it is plausible that osteopontin plays a major role in initiating chronic inflammation in *DyW* mice and recruiting immune cells to interstitial spaces during development, as well as in inhibiting early myogenesis, and paves the way for the onset of fibrosis.

In contrast to many other dystrophies, such as DMD, fibrosis appears to be a very early hallmark of MDC1A. Early increases in TGF-β signaling, a cytokine known to promote fibrosis and inhibit myofiber differentiation [51,52], implies that fibrotic signaling has begun no later than week 1. Interestingly, though collagen expression was not greatly increased until week 3, we found that periostin, another adhesive molecule of the ECM and downstream target of TGF-β, was upregulated as early as week 1. Periostin is produced by fibroblasts in non–disease scenarios and, when ablated, leads to a greatly improved fibrotic phenotype in *mdx* mice [53-55]. Thus, though collagen may not be elevated very early on, other fibrotic components of the TGF-β pathway may have already taken hold. Of note, the highest levels of periostin overexpression are coincident with the greatest increases in collagen protein. This is particularly interesting because periostin has been implicated in promoting collagen fibrillogenesis [56].

Further, we found that, at week 1, there was a large upregulation of fibronectin on both the gene and protein levels. It has previously been demonstrated that macrophages are actually capable of secreting fibronectin [57,58]. It has also been shown that fibronectin acts as a substrate for the migration of fibroblasts into tissues [58]. This is of particular importance in MDC1A because it gives rise to the possibility that the initiation of fibrosis and matrix dysregulation might be a direct result of the inflammatory nature of this disease. Additionally, Ross *et al.* demonstrated that satellite cells exposed to increased levels of fibronectin display a reduced proliferative capacity [59]. Thus, in addition to the potential of inhibited myogenesis due to early TGF-β signaling and large spaces of nonfunctional muscle, the substrate on which these muscle cells lie is innately less conducive to regeneration.

We also found that MMP-2 and MMP-9, collagenases that could help compensate for fibrotic buildup by increasing protein turnover of collagen and fibronectin, were upregulated at different time points in development. TIMP-1, an inhibitor of these molecules [60], was also upregulated, and to a much larger extent than either MMP, at all time points. These results were likely caused by one of two scenarios: Either the expression and activity of MMPs was being upregulated to compensate for increased fibrosis, subsequently leading to an increase in TIMP-1 through negative feedback, or MMP gene expression was upregulated as a response to decreased MMP activity, which was supported by the much larger increases in TIMP-1 expression. Even though we cannot know which scenario is more likely without performing an additional assay, such as zymography [61], these data further show that nearly all facets of the myomatrix in these mice were dysregulated from a very early age.

Last, in recent years, the renin-angiotensin signaling system has become increasingly understood to be intimately connected with the dysregulation of inflammation, fibrosis and ECM remodeling in many disease scenarios [27,62-64]. In this study, we found that, although the angiotensin gene itself was not dysregulated in *DyW* mice, *Ace* and two of its receptors were overexpressed very early on. It has been shown previously that blocking angiotensin II signaling with losartan can greatly improve the fibrotic *DyW* phenotype [64,65]. Additionally, losartan and other angiotensin receptor blockers have been shown to reverse the buildup of fibronectin and decrease the expression of various other matricellular proteins that we have shown to be dysregulated in *DyW* mice. Although losartan has been shown to have mixed results in the context of DMD, its impressive efficacy in models of MDC1A is likely due to the early dysregulation of fibrotic pathways in laminin deficiency.

## Conclusion

In order to properly assess and treat diseases with developmentally complex, evolving pathologies, we must study them in a temporal fashion so that we can identify the relevance of various pathomechanisms at different points in the disease progression. This will allow us to identify appropriate time windows to implement therapies targeting each of these pathologies that may be critical to their efficacy. Using the *DyW* mouse model, in this study, we provide novel insight into the development of pathologies in MDC1A and reveal that, as opposed to classical dystrophies such as DMD, the dysregulation of numerous matricellular proteins is a very early signature of laminin-deficient pathology. We also demonstrate that antiapoptotic, promyogenic and antifibrotic therapies could benefit patients most if started at a very early age. The aims of further studies that might lead to better therapies for this patient population should include determination of whether interventions that target specific pathomechanisms need to be lifelong or whether they could be restricted to given time windows during disease progression.

## Competing interests

The authors declare that they have no competing interests.

## Authors’ contributions

TM conducted gene expression and immunohistochemical experiments and wrote the body of the manuscript. AK performed the Western blot analysis and ELISA assays and contributed to all other aspects of the experimental procedures. LD performed histological and immunohistochemical analyses. JY initiated this study as part of her thesis, collected tissues and performed preliminary histological and immunohistochemical analyses. AA performed immunohistochemical experiments and manuscript revisions. SG Conceptualized and designed the study and helped in drafting the manuscript. All authors read and approved the final manuscript.

## Supplementary Material

Additional file 1: Table S1 Fold Change in Gene Expression Compared to Age-Matched WT. Click here for file

Additional file 2: Figure S1 includes comprehensive protein expression normalized to age-matched WT. Click here for file
