# Correction: dysregulation of matricellular proteins is an early signature of pathology in laminin-deficient muscular dystrophy

**DOI:** 10.1186/2044-5040-4-17

**Published:** 2014-10-02

**Authors:** Thomas Mehuron, Ajay Kumar, Lina Duarte, Jenny Yamauchi, Anthony Accorsi, Mahasweta Girgenrath

**Affiliations:** 1 Department of Health Sciences, Boston University, 635 Commonwealth Avenue, Boston, MA 02215, USA

## Correction

After publication of this work [1], we noted that while we were initially going to include muscle function tests in this study, as our manuscript evolved and we decided to include earlier time points for characterization, we decided to remove it because pups younger than 2 weeks cannot be measured. While we removed this from the results, we missed retracting them from the methods section of the abstract. Therefore, the methods section of the abstract should instead read:

## Methods

We sought out to examine the dysregulation of various pathways throughout early development (postnatal weeks 1–4) in the DyW mouse, the most commonly used mouse model of laminin-deficient muscular dystrophy. Gene (qRT-PCR) and protein levels (western blot, ELISA) as well as histology (H&E, picrosirius red staining) and immunohistochemistry (fibronectin, TUNEL assay) were used to assess dysregulation of matricellular proteins.
