# Supplementary material for: Dysregulation of matricellular proteins is an early signature of pathology in laminin-deficient muscular dystrophy
Source: Skelet Muscle. 2014 Jul 2;4:14. doi: 10.1186/2044-5040-4-14 (PMC4114446; doi:10.1186/2044-5040-4-14)
Supplement: Additional file 2: Figure S1 — includes comprehensive protein expression normalized to age-matched WT. [file 2044-5040-4-14-S2.pptx]

## Slide 1
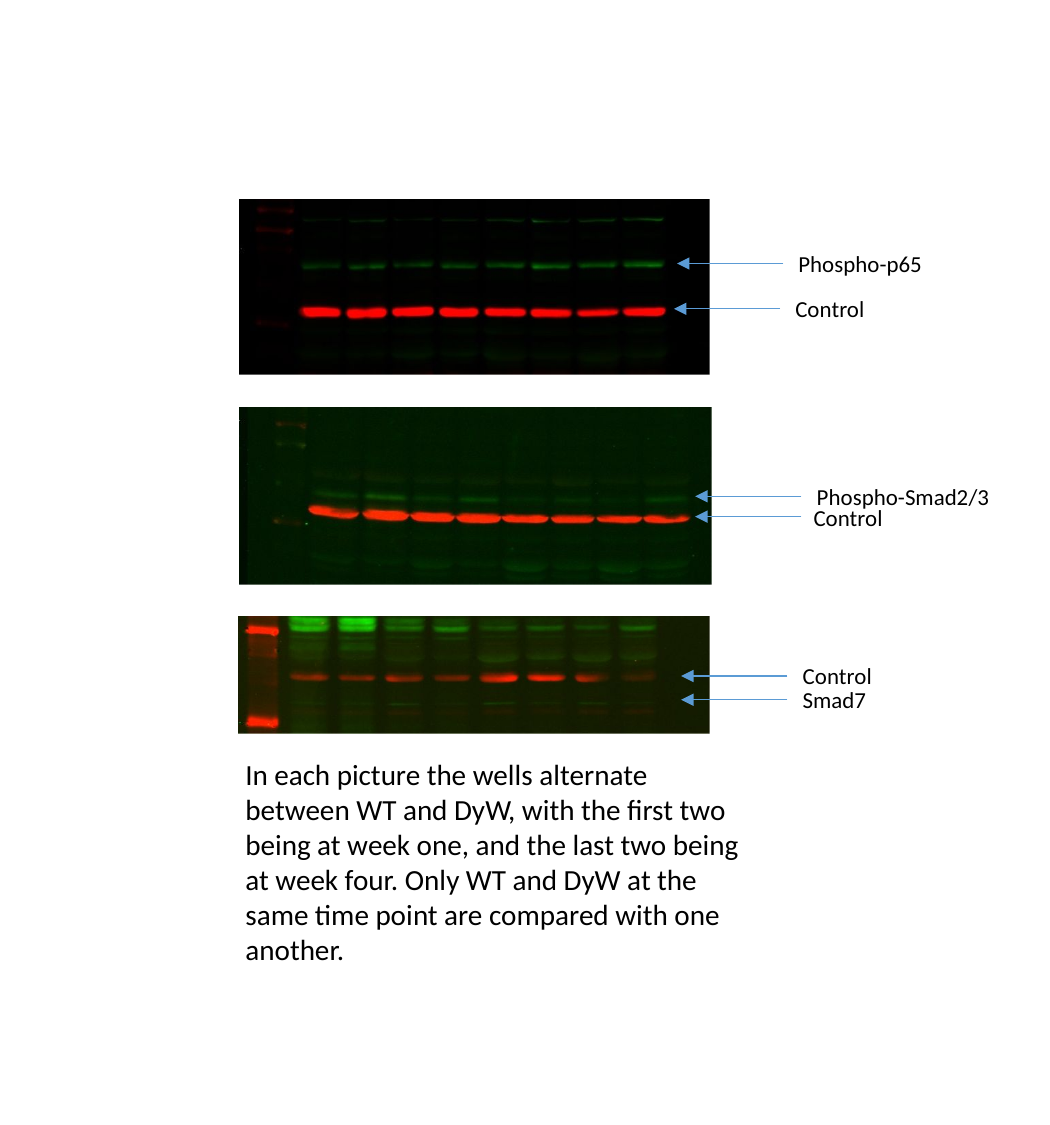

Phospho-p65
Control
Phospho-Smad2/3
Control
Control
Smad7
In each picture the wells alternate between WT and DyW, with the first two being at week one, and the last two being at week four. Only WT and DyW at the same time point are compared with one another.
